# Machine learning models to predict 30-day mortality for critical patients with myocardial infarction: a retrospective analysis from MIMIC-IV database

**DOI:** 10.3389/fcvm.2024.1368022

**Published:** 2024-09-20

**Authors:** Xuping Lin, Xi Pan, Yanfang Yang, Wencheng Yang, Xiaomeng Wang, Kaiwei Zou, Yizhang Wang, Jiaming Xiu, Pei Yu, Jin Lu, Yukun Zhao, Haichuan Lu

**Affiliations:** ^1^ Department of Spinal Surgery, Longyan First Affiliated Hospital of Fujian Medical University, Longyan, China; ^2^ Department of Pathology, Zhongshan Hospital Affiliated to Xiamen University, Xiamen, China; ^3^ Department of Cardiology, Shengli Clinical Medical College of Fujian Medical University, Fuzhou, Fujian, China; ^4^ Department of Cardiology, Longyan First Affiliated Hospital of Fujian Medical University, Longyan, China; ^5^ Department of Cardiology, The Second Affiliated Hospital, School of Medicine, Zhejiang University, Hangzhou, China; ^6^ State Key Laboratory of Transvascular Implantation Devices, Hangzhou, China; ^7^ Department of Intensive Care Unit, Longyan First Affiliated Hospital of Fujian Medical University, Longyan, China

**Keywords:** machine learning, 30-day mortality, myocardial infarction, MIMIC-IV, coronary care unit

## Abstract

**Background:**

The identification of efficient predictors for short-term mortality among patients with myocardial infarction (MI) in coronary care units (CCU) remains a challenge. This study seeks to investigate the potential of machine learning (ML) to improve risk prediction and develop a predictive model specifically tailored for 30-day mortality in critical MI patients.

**Method:**

This study focused on MI patients extracted from the Medical Information Mart for Intensive Care-IV database. The patient cohort was randomly stratified into derivation (*n* = 1,389, 70%) and validation (*n* = 595, 30%) groups. Independent risk factors were identified through eXtreme Gradient Boosting (XGBoost) and random decision forest (RDF) methodologies. Subsequently, multivariate logistic regression analysis was employed to construct predictive models. The discrimination, calibration and clinical utility were assessed utilizing metrics such as receiver operating characteristic (ROC) curve, calibration plot and decision curve analysis (DCA).

**Result:**

A total of 1,984 patients were identified (mean [SD] age, 69.4 [13.0] years; 659 [33.2%] female). The predictive performance of the XGBoost and RDF-based models demonstrated similar efficacy. Subsequently, a 30-day mortality prediction algorithm was developed using the same selected variables, and a regression model was visually represented through a nomogram. In the validation group, the nomogram (Area Under the Curve [AUC]: 0.835, 95% Confidence Interval [CI]: [0.774–0.897]) exhibited superior discriminative capability for 30-day mortality compared to the Sequential Organ Failure Assessment (SOFA) score [AUC: 0.735, 95% CI: (0.662–0.809)]. The nomogram (Accuracy: 0.914) and the SOFA score (Accuracy: 0.913) demonstrated satisfactory calibration. DCA indicated that the nomogram outperformed the SOFA score, providing a net benefit in predicting mortality.

**Conclusion:**

The ML-based predictive model demonstrated significant efficacy in forecasting 30-day mortality among MI patients admitted to the CCU. The prognostic factors identified were age, blood urea nitrogen, heart rate, pulse oximetry-derived oxygen saturation, bicarbonate, and metoprolol use. This model serves as a valuable decision-making tool for clinicians.

## Introduction

Myocardial infarction (MI) represents a widespread and severe cardiovascular ailment, characterized by substantial global morbidity and mortality. In the coronary care unit (CCU), complications such as cardiogenic shock, cardiac rupture and in-hospital cardiac arrest contribute to elevated mortality among MI patients (1–3). Robust risk prediction models are imperative for acute cardiac conditions. Currently utilized intensive care unit (ICU) risk scores, such as the Acute Physiology and Chronic Health Evaluation and Sequential Organ Failure Assessment (SOFA), play a crucial role in stratifying mortality risk (4–6). These ICU risk scores exhibit commendable discrimination, reflecting their capacity to effectively distinguish between survivors and fatalities within unselected CCU cohorts. However, it is noteworthy that calibration has been consistently suboptimal (7–10). Admission diagnoses, particularly critical care conditions like cardiac arrest, respiratory failure and shock, can significantly influence the precision of risk prediction by ICU risk scores (11, 12). The impact of admission diagnoses on the performance of risk scores in CCU patients, especially those with common cardiac diagnoses such as MI, remains underexplored. Given the grim prognosis associated with MI, an investigation into the risk factors associated with mortality becomes imperative. Accurate prediction of short-term mortality in CCU-admitted patients with MI has the potential to enhance the management and prognosis of complications related to MI.

Incorporating newer analytical methods has the potential to enhance risk prediction beyond the scope of conventional statistical approaches using existing data. Machine learning (ML) is a promising avenue for improving accuracy in predicting short-term mortality post-MI (13–15). Predominant supervised ML algorithms, including decision trees, random decision forest (RDF) and gradient boosting (GB), each possess distinct characteristics. Despite the demonstrated efficacy of multiple ML methods in the field of medicine, it is noteworthy that only a limited number of constructed models have found practical implementation in clinical settings (16). Additionally, the comparative prognostic performance of ML in predicting outcomes for patients with MI admitted to the CCU remains unknown, especially with conventional critical scores.

This study endeavors to leverage a comprehensive dataset encompassing MI patients to explore the viability and precision of ML in predicting 30-day mortality. Additionally, the objective is to develop and validate a mortality prediction model specifically tailored for MI patients admitted to the CCU utilizing the Medical Information Mart for Intensive Care-IV (MIMIC-IV) database. The anticipated outcome of this research is to contribute valuable insights that could potentially enhance medical prognosis and facilitate informed decision-making in the context of CCU-admitted MI patients.

## Method

### Data sources

The data utilized in this study were sourced from the MIMIC-IV database (https://physionet.org/content/mimiciv/0.3/), a substantial and publicly accessible resource developed and managed by the Massachusetts Institute of Technology (MIT) Computational Physiology Laboratory. Spanning the years 2008 to 2019, this database aggregates patient information derived from hourly physiological readings recorded by bedside monitors and cross-validated by ICU nurses. It encompasses a diverse and extensive cohort of ICU patients, providing comprehensive data on demographic characteristics, medical history, laboratory test and medications. Access to the database was granted upon the successful completion of the National Institutes of Health's online course, “Protecting Human Research Participants,” designed to ensure ethical research conduct involving human subjects. Specifically, individuals who passed the Collaborative Institutional Training Initiative examination, including author Lu (Certification number 10624278), were authorized to access the database. The project obtained approval from the institutional review boards of both MIT and Beth Israel Deaconess Medical Center (BIDMC), securing a waiver of informed consent for the study.

### Study population

**Figure 1:**
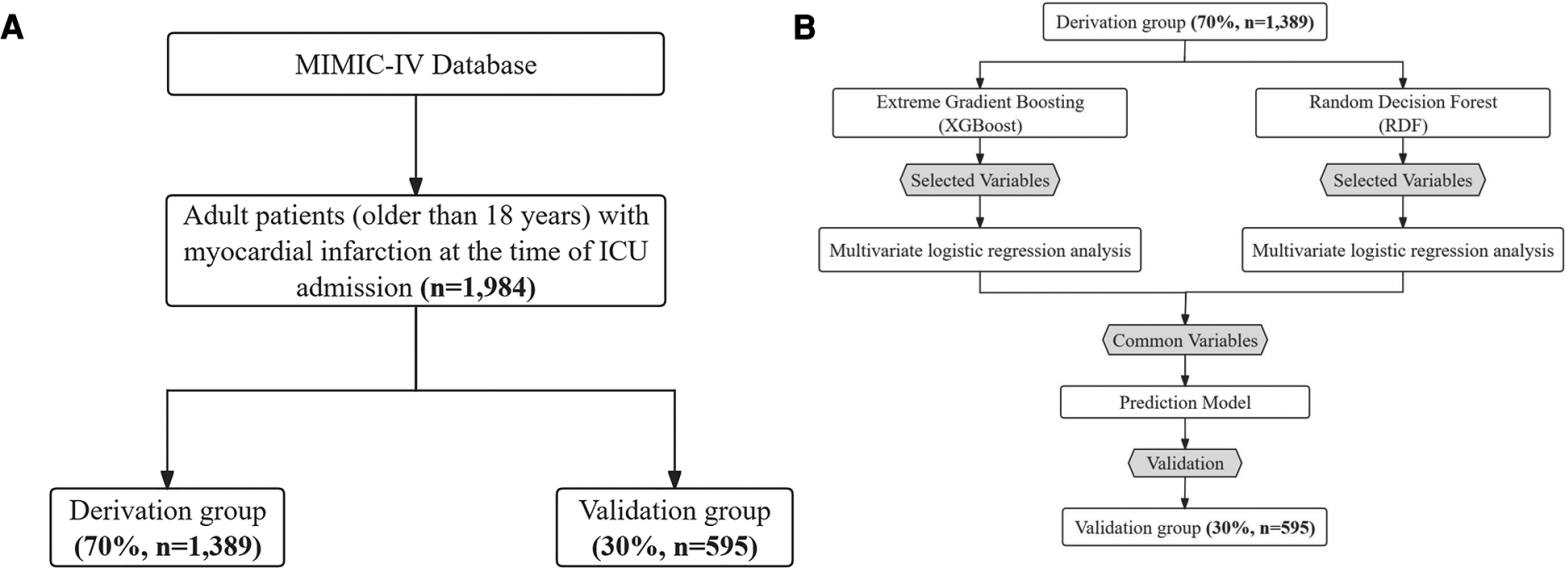
**(A)** Patient flow diagram. MIMIC-IV, medical information mart for intensive care-IV; ICU, intensive care unit. **(B)** Model development flowchart.

The MIMIC-IV database includes comprehensive and high-quality data of critically ill patients admitted to the ICU at the Beth Israel Deaconess Medical Center between 2008 and 2019. The selected population must be adults (age ≥ 18 years) diagnosed with MI, verified by manual review of ICD-9/10 codes, and with a first-time CCU admission. ICD-9 and -10 codes were documented for specific disease diagnosis in the MIMIC-IV database. We used the following ICD codes to define and extract MI patients: ICD-9: 410.0 (410.00, 410.01, and 410.02), 410.1 (410.10, 410.11, and 410.12), 410.2 (410.20, 410.21, and 410.22), 410.3 (410.30, 410.31, and 410.32), 410.4 (410.40, 410.41, and 410.42), 410.5 (410.50, 410.51, and 410.52), 410.6 (410.60, 410.61, and 410.62), 410.7 (410.70, 410.71, and 410.72), 410.8 (410.80, 410.81, and 410.82), and 410.9 (410.90, 410.91, and 410.92), and ICD-10: I21.0 (I21.01, I21.02, and I21.09), I21.1 (I21.11 and I21.19), I21.2 (I21.21 and I21.29), I21.3, and I21.4. This yielded 4,139 patients with MI from the first admission, which were then merged based on their hospital admission number. Finally, 1,984 critically ill MI patients were included. The patient selection process, outlined in Figure 1A, delineates the sequential steps leading to the final cohort of 1,984 patients extracted from the MIMIC-IV database. Rigorous measures were undertaken to preserve patient privacy, necessitating the removal of all personally identifiable information from the analytical dataset.

### Data extraction

We collected the following data, considering their clinical relevance and overall availability when patients were admitted: Demographic characteristics consisted of age, gender, race, systolic blood pressure (SBP), diastolic blood pressure (DBP), mean blood pressure (MBP), heart rate, temperature, pulse oximetry-derived oxygen saturation (SpO_2_) and glucose. Medical history comprised congestive heart failure (CHF), peripheral vascular disease (PVD), cerebrovascular disease (CVD), chronic obstructive pulmonary disease (COPD), diabetes mellitus (DM), renal disease, coronary artery bypass grafting (CABG), percutaneous coronary Intervention (PCI) and continuous renal replacement therapy (CRRT). Laboratory tests included blood urea nitrogen (BUN), hematocrit, hemoglobin, platelet, white blood cell count (WBC), red blood cell count (RBC), mean corpuscular hemoglobin (MCH), mean corpuscular hemoglobin concentration (MCHC), mean corpuscular volume (MCV), red blood cell distribution width (RDW), neutrophils, lymphocytes, international normalized ratio (INR), prothrombin time (PT), bicarbonate, potassium, calcium, sodium, chloride, anion gap and creatinine (17). Medications were also collected, including aspirin, clopidogrel, statin, metoprolol and vasoactive agents. Laboratory parameters were systematically collected within the initial 24 h of admission to the CCU, adhering to the procedures outlined by Jin Lu et al. in 2022 (18). The documentation of specific diseases using ICD-9/10 codes was carried out by hospital staff during patients’ discharge, and this information was extracted from the database through the utilization of Structure Query Language (SQL).

### Outcomes and definitions

The primary outcome of the study was all-cause mortality within 30 days of the date of admission. Monitoring of this endpoint event will involve extracting the survival status within 30 days post-admission for ICU patients from the MIMIC-IV database.

### Missing data handling

The MIMIC-IV database frequently contains variables with missing data. However, excluding patients with incomplete data introduces inherent bias into the study. Therefore, imputation becomes a critical step in data pre-processing. Variables with missing values surpassing 30% were excluded from the analysis. For the remaining missing data, imputation was performed using the mice package with random forests in R, a method known as Multivariate Imputation by Chained Equations (MICE) in R.

### Model development

A comprehensive set of 45 demographic, clinical, biochemical variables and medications were identified as potential predictors through a thorough review of current literature, expert knowledge and consideration of their applicability in clinical practice. The Extreme Gradient Boosting (XGBoost) and RDF algorithms were employed to discern the contributions of each predictor, facilitating the selection of the most relevant variables (19). From the results, the top 15 variables were chosen for subsequent analysis. To identify independent risk factors associated with 30-day mortality, univariate logistic regression analysis was conducted to assess the significance of variables selected by each method in the derivation group. Variables demonstrating a significant association with 30-day mortality were considered potential candidates for inclusion in the multivariate logistic regression model, as depicted in Figure 1B.

### SOFA score

The SOFA score, in existence for over 25 years, was devised as a succinct method for assessing and monitoring organ dysfunction in critically ill patients. Swiftly gaining prominence, the SOFA score has become a cornerstone in adult intensive care settings, finding widespread use in both clinical applications and research endeavors (20, 21).

### Displaying risk assessment using nomogram

The predictive model is designed to provide estimates of the probability or risk associated with the prospective occurrence of a specific outcome or event in individuals who are at risk of experiencing such an event. Within this framework, a nomogram serves as a visual and intuitive tool for calculating risk scores across various outcomes. By presenting a tangible representation, nomograms play a pivotal role in guiding the selection of appropriate interventions or treatments tailored to an individual's risk profile.

### Discrimination assessment

The dataset underwent random allocation into derivation (*n* = 1,389, 70%) and validation (*n* = 595, 30%) groups. Receiver Operating Characteristic (ROC) curve analysis was employed as a metric for optimizing model parameters. Discrimination performance was evaluated through the calculation of the Area Under the ROC Curve (AUC), a metric that ranges from 0.5 to 1.0. Higher AUC values signify an enhanced discriminatory ability of the model.

### Calibration assessment

Calibration characterizes the concordance between the anticipated and observed probabilities of 30-day mortality. To gauge the model's goodness-of-fit, the Hosmer-Lemeshow chi-square test was employed. This statistical test assesses the adequacy of fit between the observed and predicted outcomes from the model.

### Decision curve analysis

Decision Curve Analysis (DCA) (22) was employed to assess and compare the clinical net benefits associated with the models. The performance of the model was comprehensively evaluated by computing various metrics, encompassing AUC, sensitivity, specificity, positive predictive value, negative predictive value, positive likelihood ratio, negative likelihood ratio and accuracy. These metrics collectively offer insights into the model's discriminative ability, diagnostic accuracy and predictive value.

### Statistical analysis

To evaluate the normality of continuous variables, a normality test was executed. Continuous variables exhibiting a normal distribution were expressed as mean ± standard deviation, while those with a non-normal distribution were represented as median [interquartile range (IQR)]. Categorical data were presented as numbers (percent). Group comparisons for normally distributed continuous data employed Student's *t*-test, whereas the Kruskal–Wallis test was utilized for non-normally distributed data. Categorical data were compared using either *χ*2 or Fisher's exact test, facilitated by the tableone package in R 4.3.1. The risk score for each predictor was computed based on the beta (*β*) coefficient of the re-evaluated model (23). A two-tailed *p*-value of <0.05 indicated statistical significance in all analyses. The entire analysis was conducted using R software (V.4.3.1; https://www. R-project.org).

## Result

### Patient characteristics

Table 1 presents a comprehensive comparison of demographics and variables between deceased and surviving patients during hospitalization. The mean age of the cohort was 69.4 ± 13.0 years, with females constituting 33.2% (659 individuals). Deceased patients exhibited advanced age, a higher proportion of females, elevated heart rates and increased glucose levels in comparison to survivors. Furthermore, individuals who succumbed to their condition demonstrated a higher incidence of complications, including CHF and renal disease, while being less likely to undergo CABG and PCI in contrast to their surviving counterparts. Additionally, non-surviving patients exhibited lower lymphocyte counts and bicarbonate levels. Conversely, BUN, platelet count, WBC, MCV, neutrophil percentage, potassium levels and the proportion of vasoactive agent use were significantly higher among non-survivors. Factors such as SBP, MBP, temperature, SpO_2_, calcium levels and the proportion of metoprolol use were lower in deceased patients. Notably, there was no statistically significant difference in hematocrit levels between the survival and non-survival groups.

**Table 1:** Characteristics of participants.

Characteristics	Overall	Survival	Death	*P*-value
	*n* = 1,984	*n* = 1,763	*n* = 221	
Demographic characteristics				
Age, year	69.4 ± 13.0	68.7 ± 13.0	75.2 ± 12.1	<0.001
Female, *n* (%)	659 (33.2)	559 (31.7)	100 (45.2)	<0.001
Race				<0.001
White, *n* (%)	1,349 (68.0)	1,238 (70.2)	111 (50.2)	
Black, *n* (%)	118 (5.9)	107 (6.1)	11 (5.0)	
Asian, *n* (%)	44 (2.2)	42 (2.4)	2 (0.9)	
Other, *n* (%)	473 (23.8)	376 (21.3)	97 (43.9)	
SBP, mmHg	120.91 ± 21.71	121.70 ± 21.38	114.57 ± 23.31	<0.001
DBP, mmHg	68.26 ± 16.70	68.62 ± 16.66	65.39 ± 16.75	0.007
MBP, mmHg	84.47 ± 16.99	85.02 ± 16.77	80.09 ± 18.12	<0.001
Heart rate, beats/minute	82.85 ± 16.39	82.10 ± 15.47	88.83 ± 21.54	<0.001
T,°C	36.56 [36.39, 36.89]	36.61 [36.39, 36.89]	36.50 [36.11, 36.83]	0.010
SPO_2_,%	98.00 [95.00, 100.00]	98.00 [96.00, 100.00]	96.00 [92.00, 99.00]	<0.001
Glucose, mg/dl	141.00 [115.00, 190.00]	137.00 [113.00, 179.00]	192.00 [140.00, 281.00]	<0.001
Medical history				
CHF, *n* (%)	914 (46.1)	780 (44.2)	134 (60.6)	<0.001
PVD, *n* (%)	230 (11.6)	190 (10.8)	40 (18.1)	0.002
CVD, *n* (%)	186 (9.4)	150 (8.5)	36 (16.3)	<0.001
COPD, *n* (%)	369 (18.6)	326 (18.5)	43 (19.5)	0.798
DM, *n* (%)	756 (38.1)	666 (37.8)	90 (40.7)	0.437
Renal disease, *n* (%)	445 (22.4)	379 (21.5)	66 (29.9)	0.006
CABG, *n* (%)	702 (35.4)	677 (38.4)	25 (11.3)	<0.001
PCI, *n* (%)	888 (44.8)	804 (45.6)	84 (38.0)	0.039
CRRT, *n* (%)	67 (3.4)	35 (2.0)	32 (14.5)	<0.001
Laboratory tests				
BUN, mg/dl	19.00 [15.00, 27.00]	18.00 [14.00, 25.00]	29.00 [20.00, 41.00]	<0.001
Hematocrit,%	35.97 ± 7.13	35.95 ± 7.15	36.18 ± 7.00	0.653
Hemoglobin, g/dl	11.90 ± 2.52	11.93 ± 2.54	11.65 ± 2.28	0.117
Platelet, 10^9^/l	207.00 [159.00, 263.25]	205.00 [159.00, 259.00]	228.00 [169.00, 288.00]	0.002
WBC, 10^9^/l	11.20 [8.50, 14.70]	10.90 [8.35, 14.30]	13.90 [10.40, 18.00]	<0.001
RBC, 10^12^/l	3.97 ± 0.85	3.98 ± 0.85	3.89 ± 0.79	0.161
MCH, pg	30.10 ± 2.30	30.11 ± 2.31	30.09 ± 2.26	0.926
MCHC,%	33.03 ± 1.53	33.14 ± 1.50	32.20 ± 1.51	<0.001
MCV, fl	91.21 ± 6.27	90.91 ± 6.11	93.55 ± 7.02	<0.001
RDW,%	13.96 ± 1.68	13.89 ± 1.63	14.51 ± 1.95	<0.001
Neutrophils,%	76.44 ± 11.72	75.88 ± 11.56	80.84 ± 12.05	<0.001
Lymphocytes,%	13.70 [8.58, 20.70]	14.40 [9.20, 21.40]	8.50 [5.70, 13.60]	<0.001
INR	1.20 [1.10, 1.40]	1.20 [1.10, 1.40]	1.30 [1.10, 1.60]	<0.001
PT, second	13.00 [11.70, 15.00]	12.90 [11.65, 14.80]	14.10 [12.50, 17.50]	<0.001
Bicarbonate, mmol/l	22.28 ± 4.04	22.65 ± 3.68	19.31 ± 5.40	<0.001
Potassium, mmol/l	4.33 ± 0.74	4.32 ± 0.70	4.43 ± 1.01	0.037
Calcium, mg/dl	8.58 ± 0.76	8.61 ± 0.71	8.27 ± 1.05	<0.001
Sodium, mmol/l	137.86 ± 4.21	137.85 ± 3.87	138.00 ± 6.31	0.619
Chloride, mmol/l	102.87 ± 5.30	103.01 ± 5.01	101.80 ± 7.13	0.001
Anion gap, mmol/l	15.68 ± 4.67	15.16 ± 4.22	19.80 ± 5.89	<0.001
Creatinine, mg/dl	1.00 [0.80, 1.40]	1.00 [0.80, 1.30]	1.40 [1.10, 2.00]	<0.001
Medications				
Aspirin, *n* (%)	1,949 (98.2)	1,744 (98.9)	205 (92.8)	<0.001
Clopidogrel, *n* (%)	985 (49.6)	865 (49.1)	120 (54.3)	0.163
Statin, *n* (%)	1,899 (95.7)	1,721 (97.6)	178 (80.5)	<0.001
Metoprolol, *n* (%)	1,748 (88.1)	1,633 (92.6)	115 (52.0)	<0.001
Vasoactive agent, *n* (%)	904 (45.6)	743 (42.1)	161 (72.9)	<0.001

The patient information came from the MIMIC IV database.

SBP, systolic blood pressure; DBP, diastolic blood pressure; MBP, mean blood pressure; T, temperature; SpO_2_, pulse oximetry-derived oxygen saturation; CHF, congestive heart failure; PVD, peripheral vascular disease; CVD, cerebrovascular disease; COPD, chronic obstructive pulmonary disease; DM, diabetes mellitus; CABG, coronary artery bypass grafting; PCI, percutaneous coronary Intervention; CRRT, continuous renal replacement therapy; BUN, blood urea nitrogen; WBC, white blood cell count; RBC, red blood cell count; MCH, mean corpuscular hemoglobin; MCHC, mean corpuscular hemoglobin concentration; MCV, mean corpuscular volume; RDW, red blood cell distribution width; INR, international normalized ratio; PT, prothrombin time.

### Selected variables

**Figure 2:**
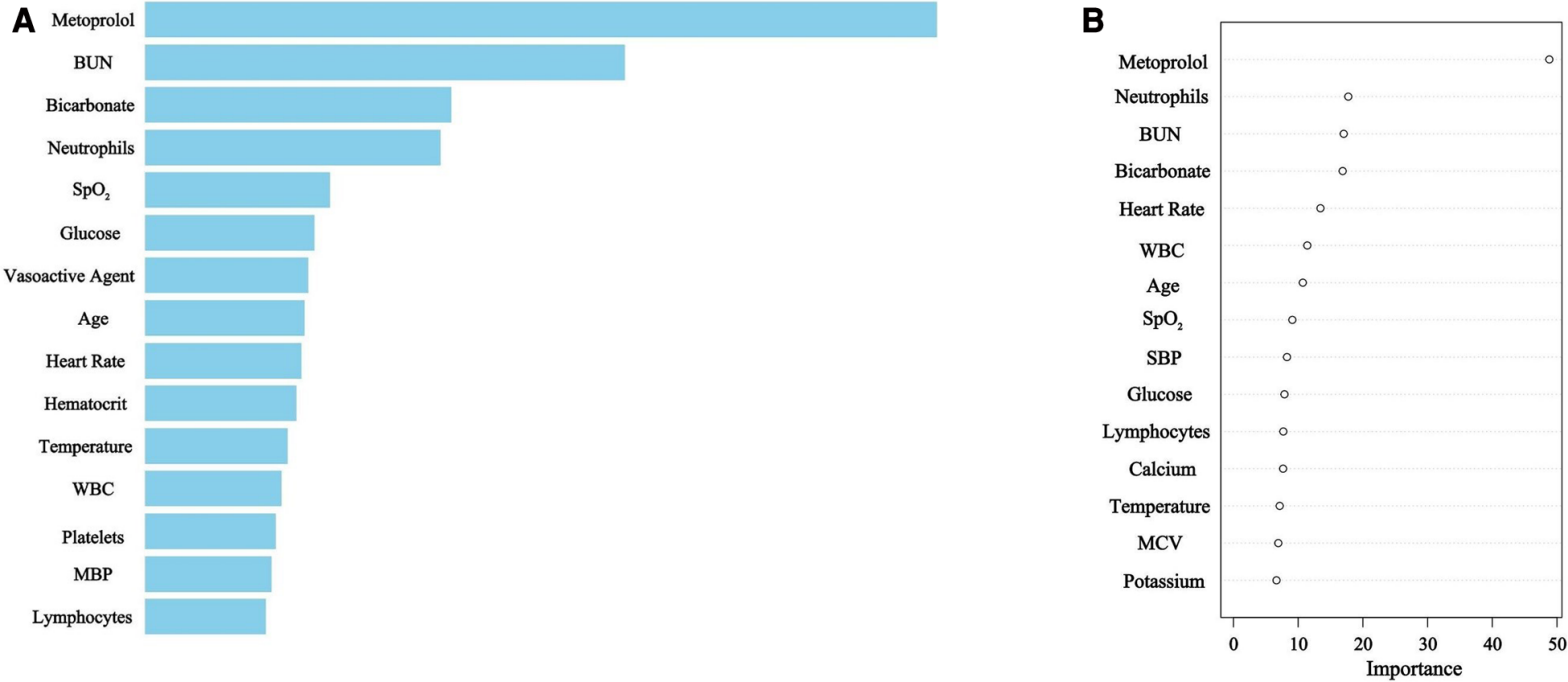
**(A)** XGBoost selected predictors. BUN, blood urea nitrogen; SpO_2_, pulse oximetry-derived oxygen saturation; WBC, white blood cell count; MBP, mean blood pressure. **(B)** Random Decision Forest selected predictors. BUN, blood urea nitrogen; WBC, white blood cell count; SpO_2_, pulse oximetry-derived oxygen saturation; SBP, systolic blood pressure; MCV, mean corpuscular volume.

Utilizing XGBoost and RDF algorithms, the set of 45 selected variables was employed to discern patients at risk of mortality within the derivation group. The relative importance of the top 15 variables utilized in the XGBoost model is depicted in Figure 2A, while Figure 2B illustrates the predictors chosen by RDF.

### Model development

**Figure 3:**
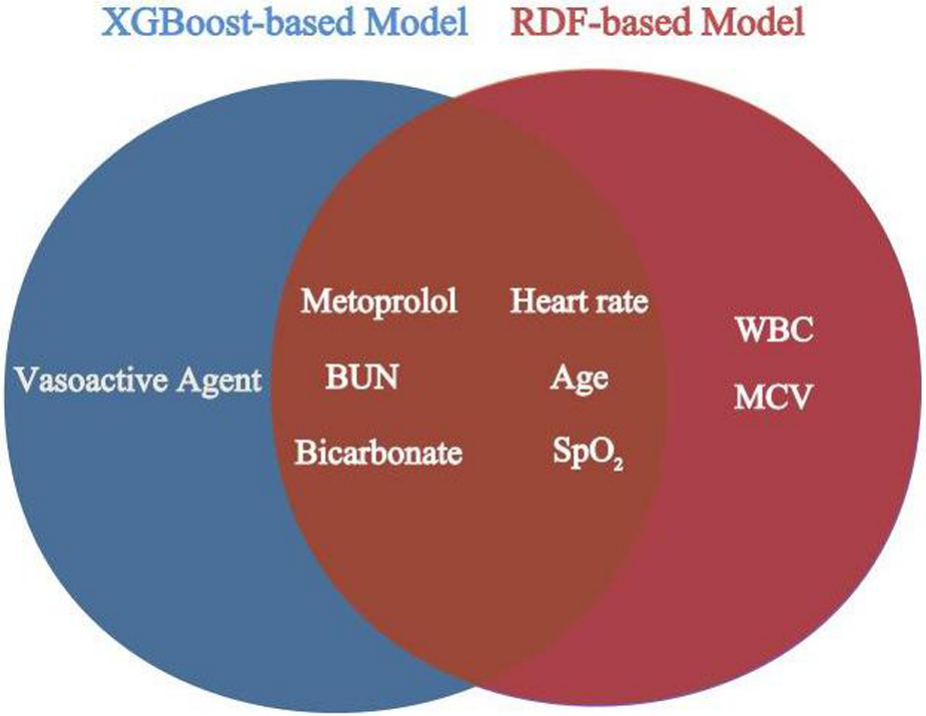
Overlapping variables of the XGBoost-based and RDF-based model were obtained in veen analysis. BUN, blood urea nitrogen; SpO_2_, pulse oximetry-derived oxygen saturation; WBC, white blood cell count; MCV, mean corpuscular volume.

For the derivation group, Tables 2, 3 present the variables selected by XGBoost and RDF, respectively, that exhibited a significant association with 30-day mortality in the univariate analysis. Variables demonstrating statistical significance in the multivariate logistic analysis were incorporated for model construction. Both the XGBoost and RDF models, exhibiting similar predictive performances (Supplementary Material) and variable counts, identified the same six variables: age, BUN, heart rate, SpO_2_, bicarbonate and metoprolol, as illustrated in Figure 3. Subsequently, a 30-day mortality prediction algorithm (PA) was formulated employing the selected variables as follows: log odds of mortality = 5.69633−2.03479 × Metoprolol−0.07936 × SpO_2_ + 0.01442 ×  BUN + 0.02831 × Age—0.10985 × Bicarbonate + 0.01688 × Heart rate.

**Table 2:** Univariate and multivariate logistic regression analysis variables screened by extreme gradient boosting in the derivation group.

	Univariate analysis			Multivariate analysis		
	OR	95%CI	*P* value	OR	95%CI	*P* value
Metoprolol	0.09	0.06–0.12	<0.001	0.16	0.10–0.25	<0.001*
Blood urea nitrogen, mg/dl	1.03	1.02–1.04	<0.001	1.02	1.01–1.03	<0.001*
Bicarbonate, mmol/l	0.83	0.80–0.86	<0.001	0.94	0.89–0.98	0.006*
Neutrophils,%	1.05	1.03–1.07	<0.001	1.02	0.98–1.07	0.258
SpO_2_,%	0.89	0.87–0.92	<0.001	0.93	0.89–0.97	0.001*
Glucose, mg/dl	1.01	1.01–1.01	<0.001	1.00	1.00–1.00	0.223
Vasoactive agent	3.68	2.70–5.03	<0.001	2.26	1.47–3.52	<0.001*
Age, years	1.04	1.03–1.06	<0.001	1.03	1.02–1.05	<0.001*
Heart rate, beats/minute	1.02	1.02–1.03	<0.001	1.01	1.00–1.02	0.015*
Hematocrit,%	1.01	0.99–1.02	0.653	1.03	1.00–1.06	0.066
Temperature,°C	0.65	0.56–0.76	<0.001	0.88	0.71–1.09	0.256
WBC, 10^9^/l	1.07	1.05–1.09	<0.001	1.01	0.99–1.03	0.203
Platelets, 10^9^/l	1.00	1.00–1.00	0.023	1.00	1.00–1.00	0.911
MBP, mmHg	0.98	0.97–0.99	<0.001	1.00	0.98–1.01	0.372
Lymphocytes,%	0.93	0.92–0.95	<0.001	1.00	0.96–1.04	0.954

SpO_2_, pulse oximetry-derived oxygen saturation; WBC, white blood cell count; MBP, mean blood pressure.

Asterisk symbol (*) indicates *p*-value <0.05, meaning a statistically meaningful difference.

**Table 3:** Univariate and multivariate logistic regression analysis variables screened by random decision forest in the derivation group.

	Univariate analysis			Multivariate analysis		
	OR	95%CI	*P* value	OR	95%CI	*P* value
Metoprolol	0.09	0.06–0.12	<0.001	0.14	0.09–0.22	<0.001*
Neutrophils,%	1.05	1.03–1.07	<0.001	1.02	0.98–1.06	0.302
Blood urea nitrogen, mg/dl	1.03	1.02–1.04	<0.001	1.02	1.01–1.03	0.001*
Bicarbonate, mmol/l	0.83	0.80–0.86	<0.001	0.94	0.89–0.99	0.012*
Heart rate, beats/minute	1.02	1.02–1.03	<0.001	1.01	1.00–1.02	0.014*
WBC, 10^9^/l	1.07	1.05–1.09	<0.001	1.02	1.00–1.04	0.019*
Age, years	1.04	1.03–1.06	<0.001	1.03	1.01–1.05	0.001*
SpO_2_,%	0.89	0.87–0.92	<0.001	0.93	0.89–0.97	0.002*
SBP, mmHg	0.98	0.98–0.99	<0.001	1.00	0.99–1.00	0.333
Glucose, mg/dl	1.01	1.01–1.01	<0.001	1.00	1.00–1.00	0.075
Lymphocytes,%	0.93	0.92–0.95	<0.001	0.99	0.95–1.03	0.759
Calcium, mg/dl	0.58	0.49–0.70	<0.001	0.84	0.65–1.09	0.184
Temperature,°C	0.65	0.56–0.76	<0.001	0.87	0.69–1.08	0.222
MCV, fl	1.07	1.05–1.10	<0.001	1.04	1.01–1.07	0.010*
Potassium, mmol/l	1.20	1.01–1.43	0.037	0.84	0.64–1.08	0.180

WBC, white blood cell count; SpO_2_, pulse oximetry-derived oxygen saturation; SBP, systolic blood pressure; MCV, mean corpuscular volume.

Asterisk symbol (*) indicates *p*-value <0.05, meaning a statistically meaningful difference.

### Model building

**Figure 4:**
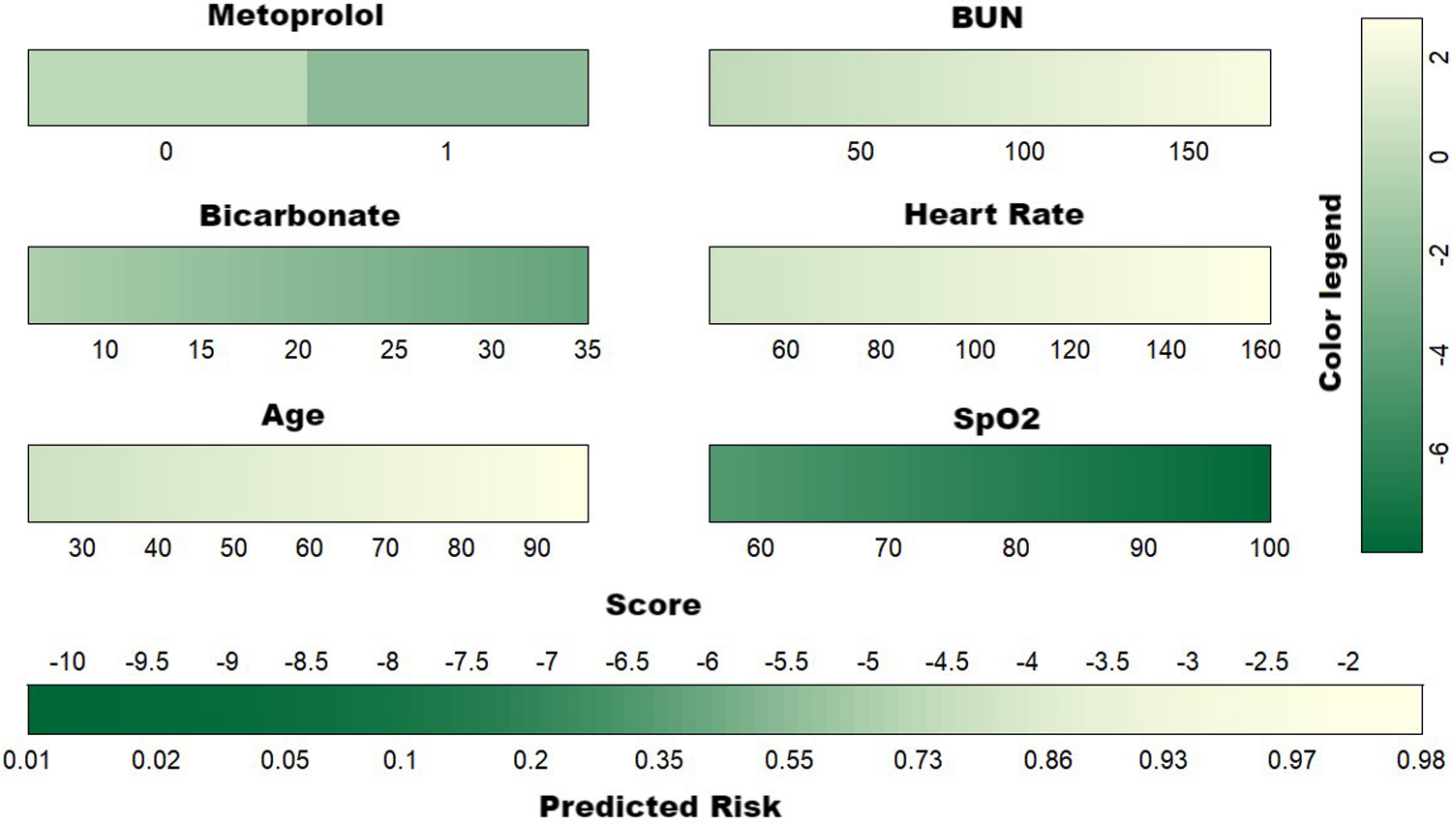
Developed nomogram for the risk of 30-day mortality. BUN, blood urea nitrogen; SpO_2_, pulse oximetry-derived oxygen saturation.

Given the comparable predictive performance and the identical number of variables in both the XGBoost and RDF models, the final predictive model was constructed using the same set of six variables. This model is visually represented by the nomogram in Figure 4.

### Model comparison

**Figure 5:**
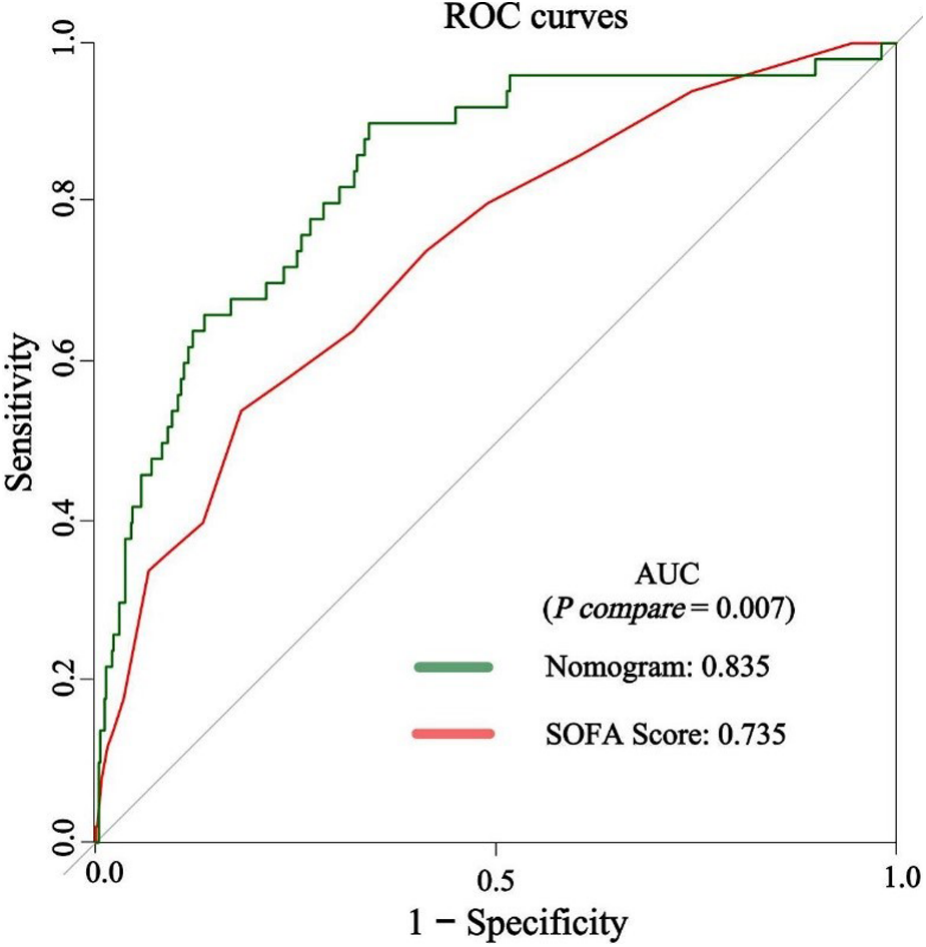
The discrimination performance of the nomogram and SOFA score in the validation group. ROC, receiver operating characteristic; AUC, area under the curve; SOFA, sequential organ failure assessment.

**Figure 6:**
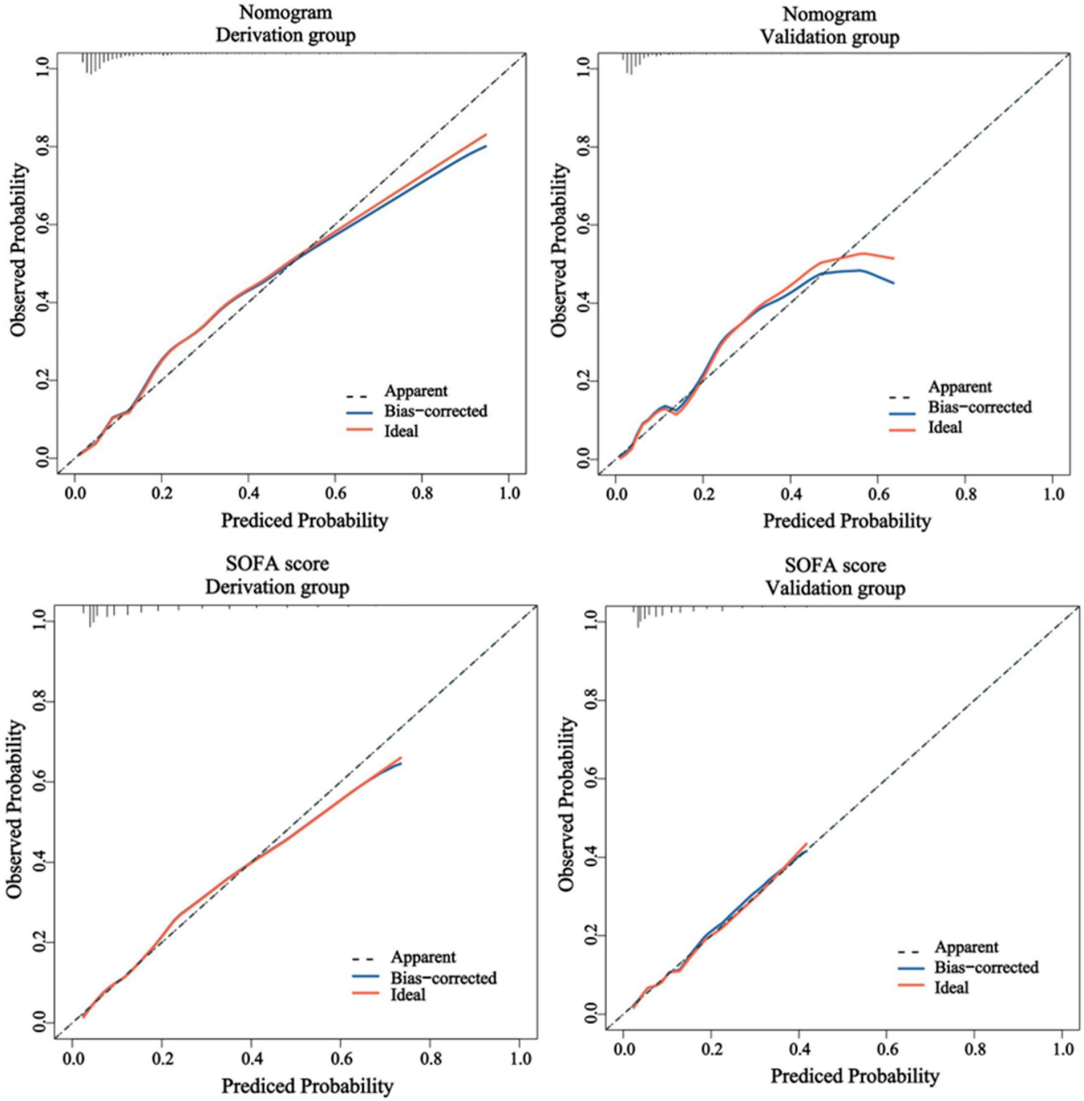
The calibration performance of the nomogram and SOFA score in the derivation and validation group. SOFA, sequential organ failure assessment.

**Figure 7:**
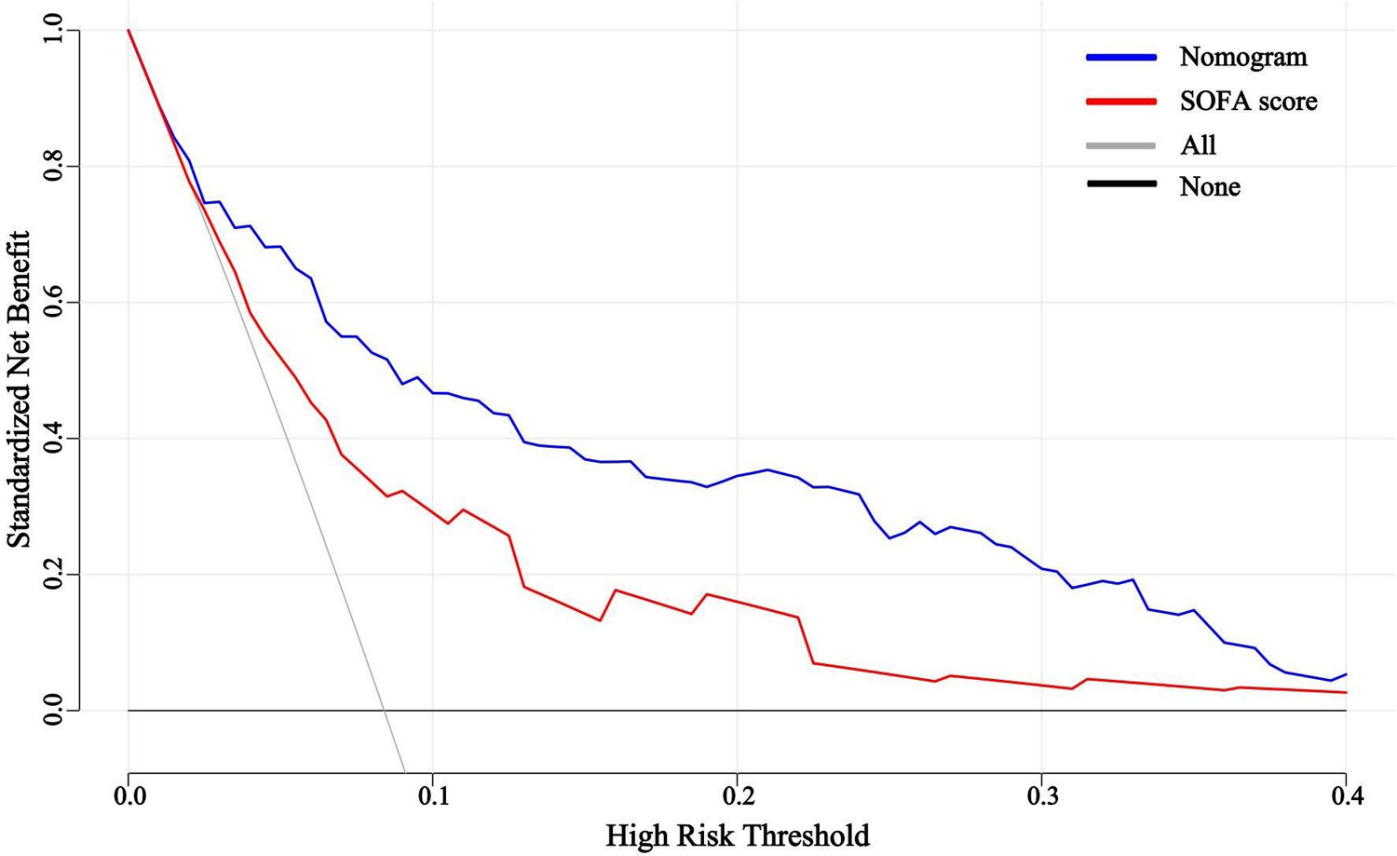
Decision curve analysis for the nomogram and SOFA score in the validation group. SOFA, sequential organ failure assessment.

The predictive efficacy of the nomogram and SOFA score models was evaluated by comparing the ROC curves of the two models (Figure 5). The calculated area under the ROC curve values for the nomogram and the SOFA score model were 0.835 (95%CI 0.774–0.897) and 0.735 (95%CI: 0.662–0.809) in the validation group, respectively. Figure 6 illustrates the calibration of both models in the derivation and validation groups, indicating robust concordance performance in both cohorts. The DCA for the nomogram and SOFA score is presented in Figure 7. Both models demonstrated a higher net benefit than either the “treat all” or “treat none” strategy, with the nomogram consistently improving the net benefit for predicting 30-day mortality compared to the SOFA score. The predictive performance of the nomogram was significantly superior to that of the SOFA risk score (*p* < 0.05), as summarized in Table 4.

**Table 4:** Pairwise comparison of prediction effectiveness for the nomogram and SOFA score.

Models	Nomogram	SOFA score
ROC area	0.835	0.735
95% CI lower	0.774	0.662
95% CI upper	0.897	0.809
Specificity	0.659	0.818
Sensitivity	0.900	0.540
Accuracy	0.914	0.913
Positive-LR	2.637	2.973
Negative-LR	0.152	0.562
Positive-PV	0.195	0.214
Negative-PV	0.986	0.951

SOFA, sequential organ failure assessment; ROC, receiver operating characteristic; CI, confidence interval; LR, likelihood ratio; PV, predictive value.

## Discussion

By leveraging data extracted from the MIMIC-IV database, we applied ML techniques, specifically XGBoost and RDF algorithms, to discern independent risk factors among critically ill patients with MI. Additionally, we pioneered the development of a 30-day mortality prediction model, aiming to offer valuable insights for clinical decision-making in advanced management strategies.

In recent years, ML has garnered widespread attention for its applications in risk prediction and disease screening, exhibiting remarkable performance. Lee et al. successfully developed an ML model for predicting 1-year mortality, showcasing excellent discriminatory ability, superior performance and good calibration (24). Oliveira et al. demonstrated the valuable role of ML methods in clinical decision-making for MI patients (14). Moreover, existing survival scoring systems following ICU admission, such as the SOFA score, have demonstrated limited efficacy, particularly in the context of MI admission. In a real-world cohort of patients diagnosed with cardiogenic shock, prevailing risk scores exhibited modest prognostic accuracy without clear indications of superiority. Further exploration is imperative to enhance the discriminative capacities of existing models or develop novel methodologies (25). Hence, this study leveraged XGBoost and RDF methodologies to uncover the intricate relationship between poor prognosis and clinical variables. Beyond conventional risk factors, XGBoost and RDF ML methods identified several novel factors, highlighting ML's advantage in enhancing classification accuracy and identification efficiency.

The most prominent feature identified by ML methods was metoprolol. Our study reveals a correlation between metoprolol administration and the prognosis of critical MI patients. Specifically, in patients with ST-elevation myocardial infarction (STEMI) undergoing primary PCI, early intravenous metoprolol administration before reperfusion has been associated with higher long-term Left Ventricular Ejection Fractions (LVEF), reduced incidence of severe left ventricular (LV) systolic dysfunction, fewer indications for implantable cardioverter defibrillator (ICD), and decreased admissions for HF (26). Early-blocker therapy has been widely recommended as an integral component of emergency treatment for suspected MI (27, 28). However, ongoing ambiguity regarding the appropriate use of intravenous-blocker therapy has persisted for several years (29–31). Our model illustrates that metoprolol significantly contributes to predicting the 30-day mortality risk in patients with critical MI.

In alignment with previous studies, our research identified the acid–base balance as a robust prognostic predictor. Zhu et al. demonstrated that the L/A ratio, bicarbonate concentration and hemoglobin level possess predictive value for 30-day mortality in patients with MI (32). Furthermore, lower serum bicarbonate levels upon admission were found to independently predict mortality in a substantial cohort of consecutive patients with cardiogenic shock hospitalized in the CCU. With the availability of point-of-care blood gas and electrolyte analyzers in the CCU, we propose that baseline serum bicarbonate levels could serve as an additional biomarker for risk identification and stratification in critical MI patients.

BUN levels also exerted a significant impact on the total point score in our model, with elevated BUN levels associated with increased 30-day mortality. The present findings align with previous studies, suggesting that a higher BUN/Cr ratio is linked to an increased risk of in-hospital mortality in patients with non-STEMI (33). Moreover, elevated levels of BUN and BUN/creatinine ratio upon admission have been identified as independent predictors of long-term mortality in patients diagnosed with STEMI (34). These consistent findings underscore the relevance of incorporating BUN levels into prognostic models for predicting the outcomes of patients with MI.

In addition to metoprolol, bicarbonate and BUN, other variables such as age and SpO_2_ hold relative importance in stratifying a patient's risk. Age is universally acknowledged as a critical risk factor for poor prognosis in CAD, and the mortality risk in MI patients escalates with advancing age (35). Moreover, with increasing age, bodily functions deteriorate, and physiological compensatory functions diminish, all of which contribute to an elevated risk of MI events (36, 37). Meanwhile, low-normal baseline oxygen saturation or the onset of hypoxemia has been identified as an independent indicator of poor prognosis (38). For patients experiencing hypoxia, personalized oxygen treatment guided by dynamic oxygen saturation is recommended.

### Limitation

Nevertheless, this study has several limitations. Firstly, the decision to admit patients to a CCU may be influenced by various factors, including practitioner judgment, institutional policies and hospital capacity, potentially introducing bias to our prediction scores. In addition, certain variables such as insurance status, marital status, troponins and natriuretic peptides were not included in the study, which may have impacted the accuracy of the results. We fully recognize the critical role of biomarkers such as troponins and natriuretic peptides in the diagnosis and treatment of myocardial infarction, and we acknowledge their absence as a significant limitation of our study. This also reflects the constraints of the database utilized, where these markers were missing at a rate exceeding 30%, precluding further in-depth analysis. Secondly, the retrospective nature of this research may introduce selective bias, and we rigorously included eligible patients according to predefined criteria to mitigate this potential bias. Thirdly, being a single-center study, the sample size was relatively modest. While the robustness of our risk model was rigorously tested through internal validation using bootstrap testing, the generalizability of these study results to other populations remains uncertain. Lastly, our model may primarily serve to promptly recognize critical clinical situations at the bedside and might not offer additional insights into potential life-threatening pathophysiological mechanisms.

## Conclusion

We developed the 30-day mortality prediction model for CCU-admitted MI patients. This tool incorporates a concise set of routinely collected variables, facilitating ease of use at the bedside. Moreover, it can be seamlessly integrated into CCU monitoring technology, enabling automated notifications to CCU staff at various stages of the illness.

## Data Availability

The datasets presented in this study can be found in online repositories. The names of the repository/repositories and accession number(s) can be found below: MIMIC-IV database (https://physionet.org/content/mimiciv/0.3/) Certification number 10624278.
