# Supplementary material for: Machine learning models to predict 30-day mortality for critical patients with myocardial infarction: a retrospective analysis from MIMIC-IV database
Source: Front Cardiovasc Med. 2024 Sep 20;11:1368022. doi: 10.3389/fcvm.2024.1368022 (PMC11449713; doi:10.3389/fcvm.2024.1368022)
Supplement: Supplementary file 1 [file Table1.pdf]

Supplementary Table 1 Pairwise comparison of prediction effectiveness for the XGBoost-based model and RDF-based model.

Models	XGBoost-based model	RDF-based model
ROC area	0.834	0.832
95% CI lower	0.769	0.768
95% CI upper	0.900	0.897
Specificity	0.774	0.785
Sensitivity	0..780	0.780
Accuracy	0.919	0.921
Positive-LR	3.456	3.633
Negative-LR	0.284	0.280
Positive-PV	0.241	0.250
Negative-PV	0.975	0.975

Abbreviations: XGBoost, Extreme Gradient is boosting; RDF, Random decision Forest; ROC, receiver operating characteristic; CI, Confidence Interval; LR, likelihood ratio; PV, predictive value.
